# Use of Multi-Functional Flexible Micro-Sensors for *in situ* Measurement of Temperature, Voltage and Fuel Flow in a Proton Exchange Membrane Fuel Cell

**DOI:** 10.3390/s101211605

**Published:** 2010-12-20

**Authors:** Chi-Yuan Lee, Pin-Cheng Chan, Chung-Ju Lee

**Affiliations:** Department of Mechanical Engineering, Yuan Ze Fuel Cell Center, Yuan Ze University, Taoyuan, Taiwan; E-Mails: s975132@mail.yzu.edu.tw (P.-C.C.); s950848@mail.yzu.edu.tw (C.-J.L.)

**Keywords:** multi-functional flexible micro-sensors, *in situ*, PEMFC

## Abstract

Temperature, voltage and fuel flow distribution all contribute considerably to fuel cell performance. Conventional methods cannot accurately determine parameter changes inside a fuel cell. This investigation developed flexible and multi-functional micro sensors on a 40 μm-thick stainless steel foil substrate by using micro-electro-mechanical systems (MEMS) and embedded them in a proton exchange membrane fuel cell (PEMFC) to measure the temperature, voltage and flow. Users can monitor and control *in situ* the temperature, voltage and fuel flow distribution in the cell. Thereby, both fuel cell performance and lifetime can be increased.

## 1. Introduction

As the demand for new sources of energy is rapidly increasing, more research on fuel cells is being undertaken. Some critical problems are being solved. The parameters that significantly affect the performance of fuel cells are water and heat management. The temperature and voltage distributions in a membrane electrode assembly (MEA) are also important. Accordingly, *in situ* temperature, voltage and fuel flow diagnosis have become important for determining cell performance and lifetime. Cell efficiency is markedly reduced and service lifetime shortened when these parameters are outside the optimum range.

Wu [1] successfully integrated a micro temperature sensor on a PDMS. Inman [2] presents thermal sensors based on the principles of the lifetime-decay method of phosphor thermometry to measure temperatures inside a proton exchange membrane fuel cell (PEMFC). Freunberger [3] adopted the potential drop over the flow field plate and the gas diffusion layer to gain the current distribution in a fuel cell. Gagliardo [4] fabricated a polytetrafluoroethylene substrate with 10 × 10 array sensors giving additional insight into water management and fuel cell performance under a variety of conditions. A measurement signal was generated with a custom high-speed data-acquisition system and compared with a neutron image. Ma [5] developed a MEMS-based low-cost sensing platform for sensing the rate and direction of gas flow, comprising four silicon nitride cantilever beams arranged in a cruciform configuration. The flow rate is derived from the inverse of the change in the resistance signal of the flow meter when it is exposed to the sensed air stream. Shikida [6] fabricated an integrated sensor on a Ti substrate by photolithography and wet etching, with the advantage that both the thermal isolation cavity and the structure could be fabricated in a single etching process.

In reviewing other works, it becomes clear that researchers almost invariably determine fuel cell parameters either on a large scale or using *ex situ* or invasive measurements, and no integrated temperature, voltage and fuel flow sensors for *in situ* monitoring in fuel cells exist. To avoid the use of sensors that occupy too large an area of the reactant, in this investigation micro-electro-mechanical systems (MEMS) that integrate temperature, voltage and flow sensors are used to reduce the proton insulating area and stainless steel (40 μm thick) is used as a flexible substrate. The volume of these micro sensors is sufficiently small that they can be placed anywhere between the MEA and the flow channel and no support frame is required. Such micro sensors have the following advantages; (1) they are small; (2) they are highly sensitive; (3) they are flexible and strong; (4) they can be mass-produced; (5) they can be placed anywhere to make measurements, and, (6) and they can be used to make measurements *in situ* [7].

Micro-electro-mechanical systems (MEMS) have novel applications and can be integrated with micro flexible temperature, voltage and flow sensors using innovative fabrication techniques. Multi-functional flexible micro-sensors are fabricated on a stainless steel foil (40 μm) flexible substrate. Micro sensors in a fuel cell have the advantages of multi-functionality, high accuracy, high linearity, high sensitivity, extreme flexibility, mass producibility and a short response time.

## 2. Method

### 2.1. Theory of Micro Temperature Sensor

**Figure 1.**
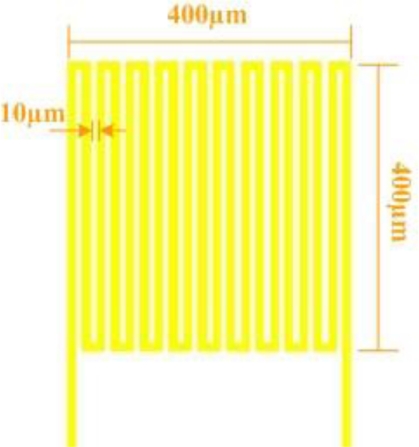
Design of a micro temperature sensor.

In this study, a resistance temperature detector (RTD) was used as a micro temperature sensor. As the environmental temperature increases, the resistance of the RTD also increases, because a metal conductor has a positive temperature coefficient (PTC) of resistance. The electrodes had serpentine micro temperature sensor structures, with a sensing area of 400 μm × 400 μm, as shown in Figure 1. As the temperature of the RTD varies, the linear relationship between the measured resistance and the change in temperature is given by:

(1) $$R_{t}=R_{i}(1+\alpha_{T}\Delta T)$$

where *R_t_* represents the resistance at the measurement temperature; *R_i_* is the resistance at a reference temperature, and α*_T_* is the sensitivity (1/°C) [8].

Equation (1) can be rewritten as:

(2) $$\alpha_{T}=\frac{R_{t}-R_{i}}{R_{i}(\Delta T)}$$

### 2.2. Theory of Micro Voltage Sensor

**Figure 2.**
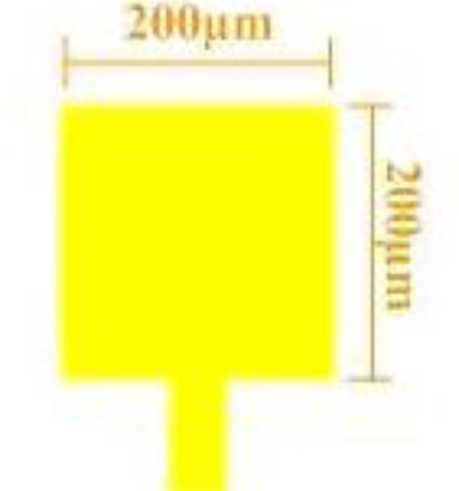
Design of a micro voltage sensor.

**Figure 3.**
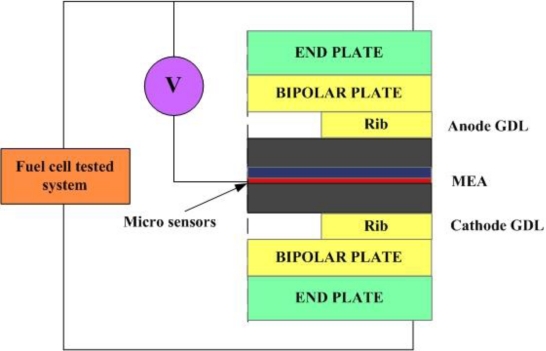
Measurement system configuration.

Based on Ohm’s law, the current through a conductor between two points is directly proportional to the potential difference or voltage across the two points, and inversely proportional to the resistance between the two points:

(3) V=I×R

where V represents the potential difference measured across the resistance (Volts); I represents the current through the resistance (Amperes), and R represents the resistance of the conductor (Ω). Figure 2 presents the design of a micro voltage sensor. The sensing area of the voltage sensor, in contact with the bipolar plate of the fuel cell, is 200 μm × 200 μm. Figure 3 schematically depicts the measurement system.

### 2.3. Theory of Micro Flow Sensor

Micro flow sensors are either thermal flow sensors or non-thermal flow sensors. Thermal flow sensors comprise: a (1) hot wire/hot film anemometer, (2) calorimetric sensor, and (3) time-of-flight flow sensor. All parts can be manufactured using MEMS technology. In a hot-wire, electrical energy is converted to thermal energy by Joule heating. Governed by the temperature coefficient of resistance of the wire material, *α*, the increase in wire temperature changes the resistance, according to King’s Law [9], the relation of thermal energy dissipation rate and flow speed is shown in Equation (4):

(4) $$\text{Q}={\text{I}}^{2}\times \text{R}=\text{I}\times \text{V}=(\text{A}+\text{B}+{\text{U}}^{\text{n}})\;\;(\text{Ts}-{\text{T}}_{0})$$

where Q represents the power that outer power supplier provided for a hot wire; U denotes the flow speed; Ts refers to the hot wire temperature; T_0_ is the flow temperature; n denotes the related coefficient of U and Q, approximately 0.5 by experiment; A represents a constant while the flow speed is 0, calorie coefficient transferred by heater; and B refers to a constant while the flow speed in a non zero, calorie coefficient is transferred by heater. Equation (4) can thus be rewritten as:

(5) $$\text{Q}=(\text{A}+\text{B}\times {\text{U}}^{0.5})\;\Delta \text{T}$$

A micro temperature sensor can be also use as a micro flow sensor [10], as shown as Figure 1.

## 3. Fabrication of Multi-Functional Flexible Micro-Sensors

In this study, multi-functional flexible micro-sensors were fabricated on a stainless steel foil substrate (SS-304, 40 μm thick), and aluminum nitride (AlN) was utilized as an insulation layer because it has excellent insulation properties.

**Figure 4.**
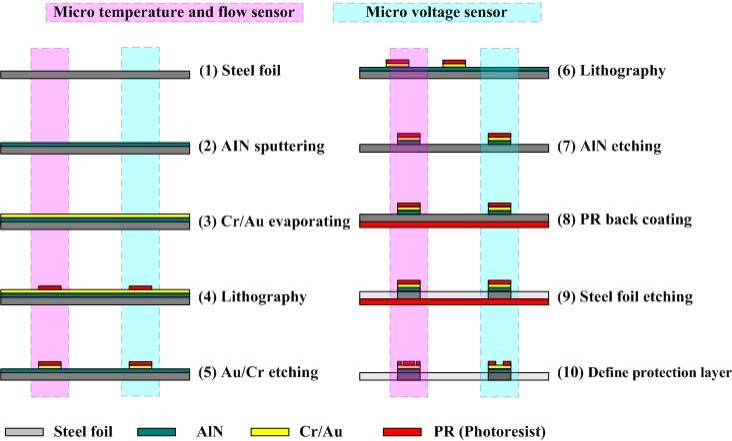
Process of fabricating multi-functional flexible micro-sensors.

**Figure 5.**
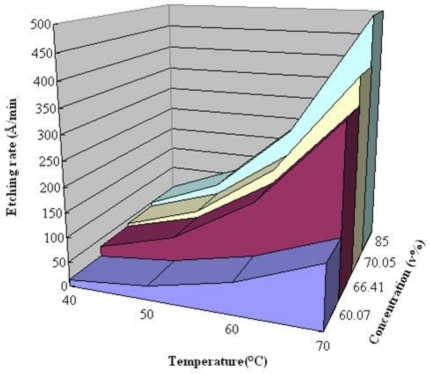
Etching rate of phosphoric acid.

**Figure 6.**
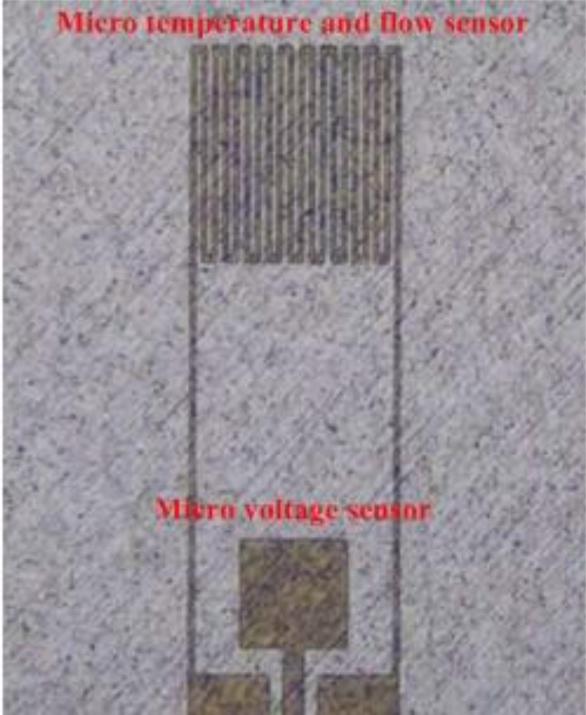
SEM image of micro temperature/ flow and voltage sensors.

Figure 4 displays the process of fabricating multi-functional flexible micro-sensors. First, sulfuric acid and hydrogen peroxide are used to clean the stainless steel foil; AlN (1 μm) is then sputtered as the bottom insulation layer. An E-beam evaporator is then used to deposit Cr (400 Å) as an adhesive layer between the AlN and Au. Evaporated Au (2,200 Å) is deposited as the sensing layer. The photoresist (PR, 3 μm) is then spin-coated and the outline of the multi-functional flexible micro-sensors is defined lithographically by wet etching. Phosphoric acid with a concentration of 85% is used as the etchant. The etching rate is around 500Å/min under a standard condition of 70 °C. Figure 5 illustrates the etching rate of temperature and concentration. The PR (5 μm) is again spin-coated as a protection layer. Finally, the stainless steel foil is etched using aqua regia [7]. Table 1 summarizes the recipe for multi-functional flexible micro-sensors, while Figure 6 displays a SEM image of the micro temperature and voltage sensors.

## 4. Results and Discussion

In the experiment, the cell temperature was set to 50 °C, 60 °C and 70 °C. Both anode and cathode were humidified at 100%RH. A constant current of 25 A was output for 30 minutes. The response time of thermocouple is 0.003 second. The gas flow rates of air and hydrogen were fixed. When the anode and cathode generated 5 A/cm^2^, the air and hydrogen flow rates had to be 820 sccm and 350 sccm, respectively. The operating conditions are shown in Table 2.

**Figure 7.**
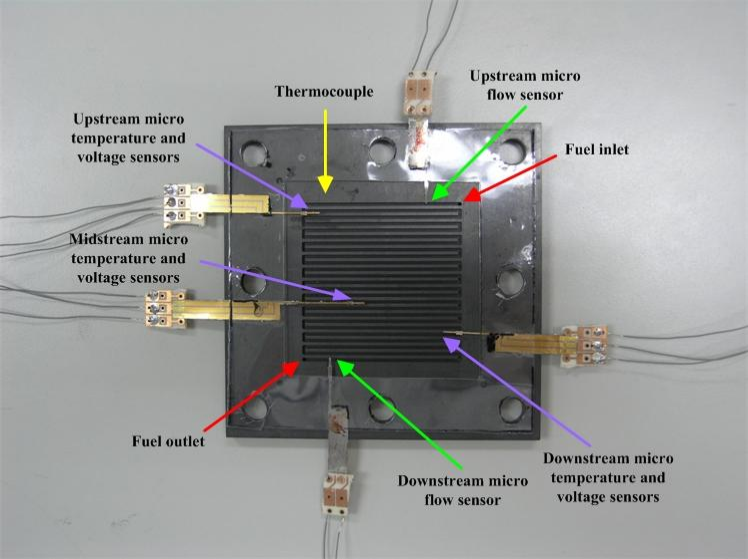
Location of flexible micro-sensors.

As Figure 7, micro temperature sensors were embedded upstream, midstream and downstream respectively. Micro voltage sensors were also embedded upstream, midstream and downstream respectively. Micro flow sensors were embedded upstream and downstream.

**Figure 8.**
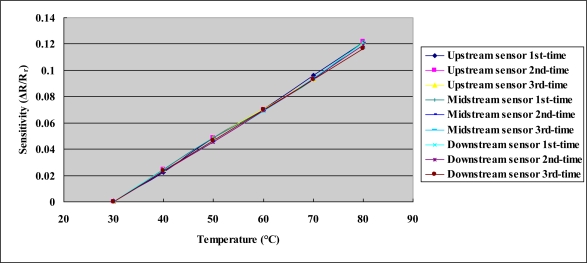
Calibration curves of the micro temperature sensors.

**Figure 9.**
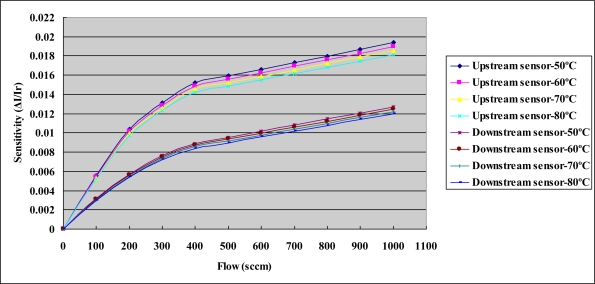
Calibration curves of the micro flow sensors.

Figure 8 depicts the calibration curves of the micro temperature sensors. Each micro temperature sensor was placed in a Hungta HT-8045A programmable temperature chamber. The resistance of the micro temperature sensor was measured using an LCR meter. The temperature measured by the micro sensor varied from 30 to 80 °C. The results reveal that the temperature was almost linearly related to resistance. In three tests, the calibration curve was linear. The sensitivity of the micro temperature sensor was 0.8 ± 0.005 Ω°C^−1^, and the accuracy was less than 0.5 °C. Figure 9 depicts the calibration curves of micro flow sensors with the flow rate varied from 0 to 1,000 mL/min.

### 4.1. Flow Monitoring

**Figure 10.**
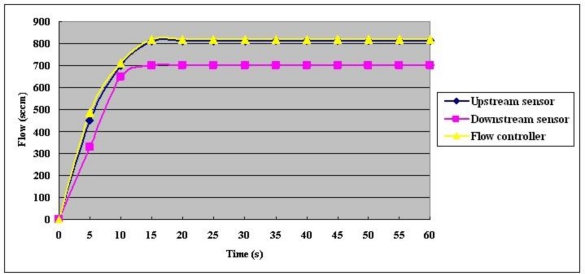
Constant current 25A test flow trend chart.

As shown in Figure 10, the inner gas flow rate reaches equilibrium after the fuel cell has been running for 15 s. Upstream and downstream air gas flow rates of 800 sccm and 700 sccm, respectively, are measured using the micro flow sensors. The difference between the upstream and downstream values is consistent with results of the leak test that was performed before the experiment.

### 4.2. Temperature Monitoring

**Figure 11.**
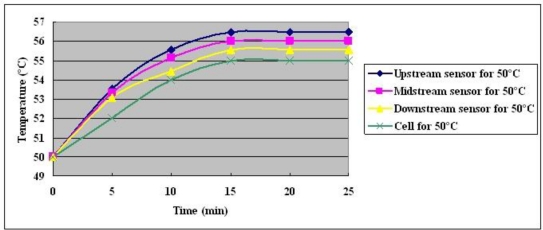
Temperature distributions at cell temperature of 50 °C with constant output current of 25 A.

**Figure 12.**
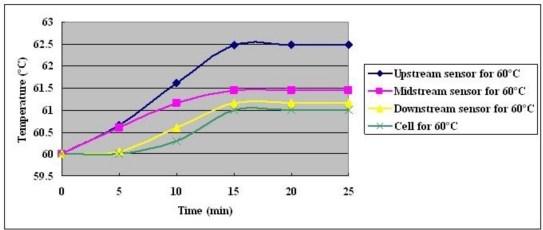
Temperature distributions at cell temperature of 60 °C with constant output current of 25 A.

**Figure 13.**
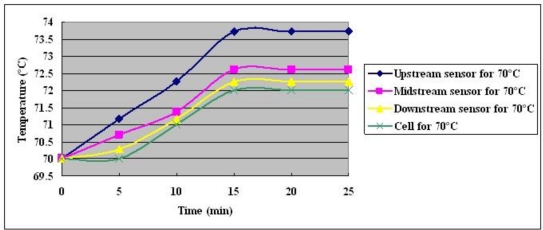
Temperature distributions at cell temperature of 70 °C with constant output current of 25 A.

The measured temperature in the fuel cell at a constant current output of 25 A for 30 minutes is shown in Figures 11–13. The following items are indicated:

1. Since sufficient fuel is available, enough gas is present to remove generated water. At three temperatures and a fixed output current of 25 A, generated water is removed in a short time, and cell performance is recovered [11].
2. The temperature that is measured in the local MEA exceeds the external temperature, which is measured using a thermocouple.
3. At three set temperatures, the upstream and midstream temperature exceed the downstream one.
4. The maximum temperature difference is 1.73 °C in upstream when the cell temperature is 70 °C.

### 4.2. Voltage Monitoring

**Figure 14.**
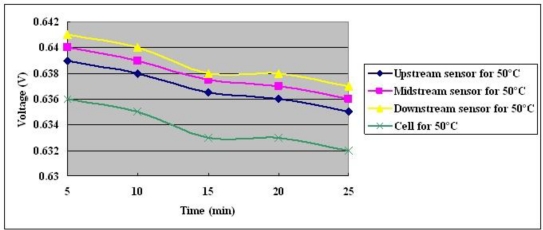
Voltage distributions at cell temperature of 50 °C with constant output current of 25 A.

**Figure 15.**
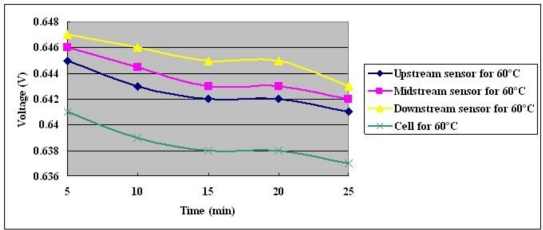
Voltage distributions at cell temperature of 60 °C with constant output current of 25 A.

**Figure 16.**
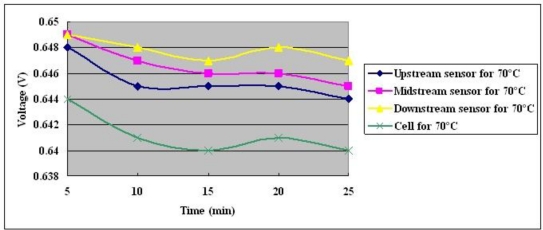
Voltage distributions at cell temperature of 70 °C with constant output current of 25 A.

The measured voltage in the fuel cell at a constant current output of 25 A for 30 minutes is shown in Figures 14–16. They indicate the following items:

1. Flooding can be eliminated rapidly when sufficient fuel is supplied.
2. The inner voltage exceeds the outer voltage.
3. The maximum difference between the inner potential and the outer potential is 7 mV at a cell temperature of 70 °C. The voltages of upstream and midstream exceed that downstream.

The fuel cell introduces electricity in membrane electrode assembly (MEA); the potential will lose from MEA to bipolar plate because of the enhancement of contact resistance. Therefore, inner voltage exceeds the outer voltage.

## 5. Conclusions

In this investigation, micro temperature, voltage and flow sensors were integrated in a multi-functional flexible micro-sensor on a 40 μm-thick stainless steel foil substrate. The micro sensors were fabricated using MEMS and can be utilized to monitor operating fuel cells in real time. A three-in-one sensor was embedded in a fuel cell for the *in situ* diagnosis of local data. The method is more precise than other invasive or *ex situ* measurement methods and provides information about the interior workings of the fuel cell.

## Figures and Tables

**Table 1.** Recipe for multi-functional flexible micro-sensors.

**Step**	**Recipe**
1	**Steel foil:** stainless steel foil substrate (SS-304 40 μm thick) H_2_SO_4_ + H_2_O_2_ (cleaning 10mins)
2	**AlN sputtering:** Ar (6.8 sccm), N_2_ (1.7 sccm), Pressure (0.23 Pa), Substrate temperature (120 °C), Power (150 W)
3	**Cr / Au evaporating:** Substrate temperature (100 °C), Background pressure (8 × 10^−7^ Torr), Cr thickness (400 Å), Au thickness (2,200 Å)
4	**Lithography:** Spin coated photoresist→ 6,000 rpm, 30 s. Soft bake→ 110 °C, 90 s. Exposure→182 mJ/cm^2^. Development→MP 2500:DI water = 5:1, Hard bake→ 110 °C, 10 mins
5	**Au / Cr etching:** Au etching (KI + I_2_,7 mins), Cr etching (Cr-7, 3 mins)
6	**Lithography:** Spin coated photoresist→ 6,000 rpm, 30 s. Soft bake→ 110 °C, 90 s. Exposure→ 182 mJ/cm^2^. Development→ MP2500:DI water = 5:1, Hard bake→ 110 °C, 10 mins
7	**AlN etching:** H_3_PO_4_ (70 °C)
8	**PR back coating:** Spin coated photoresist→ 1,000 rpm, 10 s. Hard bake→ 110 °C, 30 mins.
9	**Steel foil etching:** Aqua regia (40 °C)
10	**Define protection layer:** Spin coated photoresist→ 6,000 rpm, 30 s. Soft bake→ 110 °C, 90 s. Exposure→ 182 mJ/cm^2^. Development→ MP2500:DI water = 5:1, Hard bake→ 110 °C, 30 mins

**Table 2.** Operating conditions.

**Items**	**Conditions**

Cell temperature	50 °C, 60 °C, 70 °C
Relative humidity (%RH)	100%
H_2_ flow rate (Anode)	350 sccm (λ = 2x @ 1A cm^−2^)
Air flow rate (Cathode)	820 sccm (λ= 2x @ 1A cm^−2^)
Bipolar plate/Flow field type	Graphite/ Single-path serpentine
Flow-channel depth	1 mm
Flow-channel width	1 mm
Flow-rib width	1 mm
Reaction area	25 cm^2^
